# Neurodiversity Assessments and Management in Community Mental Health Services

**DOI:** 10.1192/bjo.2025.10377

**Published:** 2025-06-20

**Authors:** Mohammed Elsankary, Stephanie Iyamu, Nyembezi Faith Ndebele

**Affiliations:** 1 Hampshire and Isle of Wight NHS Foundation Trust, Winchester, United Kingdom; 2 Hampshire and Isle of Wight NHS Foundation Trust, Portsmouth, United Kingdom

## Abstract

**Aims:** This quality improvement project aimed to improve the experience and outcomes for neurodiverse patients accessing secondary community mental health services. It focused on reducing delays in neurodevelopmental assessments, delivering timely and evidence-based interventions, and enhancing clinician awareness and skills in identifying symptoms indicative of neurodiverse conditions, particularly in patients with co-occurring mental health needs.

By integrating these goals, the project aspired to create a more inclusive and effective care environment for neurodiverse individuals.

**Methods:** This project involved a structured approach.

Identification of Patients: Patients with symptoms suggestive of neurodiverse conditions were identified during multidisciplinary team (MDT) meetings, followed by a review of clinical records for relevant indicators, in secondary community mental health services.

Comprehensive Assessments: Each patient underwent a full psychiatric assessment, which included clinical and semi-structured interviews informed by validated tools to ensure a thorough evaluation of neurodevelopmental symptoms.

Feedback and Evaluation: Clinician feedback and patient survey were collected to assess the project’s impact on care delivery, patient engagement, and satisfaction with the services provided.

**Results:** This project achieved notable results, with 18 patients referred for neurodevelopmental assessments and 15 assessments completed. Of those, 60% received a confirmed diagnosis, with 77.78% diagnosed with ADHD and 22.22% with autism spectrum disorder (ASD). All patients received individualized interventions, including psychoeducation and tailored treatment plans. ADHD patients were monitored at 2-week, 4-week, and 8-week intervals for medication management, while ASD patients were referred for psychosocial support. Additionally, four patients used an ADHD digital support tool (SilverCloud) to manage ADHD and anxiety symptoms.

However, challenges such as comorbidities (e.g., substance misuse and PTSD) made patient engagement and follow-up more difficult. Some patients also struggled to use the digital support tool due to time limitations or personal preferences.

**Conclusion:** This project highlighted the importance of integrating neurodevelopmental assessments into secondary community mental health services. By reducing waiting, patient outcomes and satisfaction were significantly improved. Despite challenges such as comorbidities, the project highlighted the value of providing timely and personalized care for neurodiverse individuals. These findings offer valuable insights for future initiatives, stressing the need for mental health services that are both inclusive and responsive to the unique needs of neurodiverse patients.

